# Smart Self-Nourishing and Self-Healing Artificial Skin Composite Using Bionic Microvascular Containing Liquid Agent

**DOI:** 10.3390/polym14193941

**Published:** 2022-09-21

**Authors:** Xu Gao, Jun-Feng Su, Sai Wang, Peng Yang

**Affiliations:** 1 School of Material Science and Engineering, Tiangong University, Tianjin 300387, China; 2 School of Civil Engineering and Management, Guangzhou Maritime University, Guangzhou 510725, China

**Keywords:** artificial skin, self-nourishing, self-healing, microvascular

## Abstract

Artificial skin composites have attracted great interest in functional composite materials. The aim of this study was to prepare and characterize a smart artificial skin composite comprising a bionic microvascular with both self-nourishing and self-healing functions. A poly(vinyl alcohol)–glycerol–gelatin double network organic hydrogel was used as the artificial skin matrix. The hydrogel had high mechanical strength because of the strong hydrogen bond formed between the PVA and glycerol (GL). The gelatin (GEL) increased the toughness and elasticity of the hydrogel to ensure the strength of the artificial skin and fit of the interface with the body. The bionic polyvinylidene fluoride (PVDF) microvascular had excellent thermal stability and mechanical property in artificial skin. Results indicated that self-nourishing was successfully realized by liquid release through the pore structures of the bionic microvascular. The bionic microvascular healed microcracks in the artificial skin when damage occurred, based on a self-healing test.

## 1. Introduction

Skin is the first protection of the human body and is also one of the largest organs, covering the entire body. Skin has many functions [1,2]; it can control the body temperature, feel stimulation from external sources, and moisten itself by secreting sebum in normal conditions. In addition, skin can self-heal when injured [3]. Various artificial skins have been studied for the body and biomimetic bodies (biomimetic robot, artificial body). Such artificial skins are made from various materials such as polyurethane [4], graphite [4,5], nanofiber fabrics [6], and gels [7,8,9,10]; among these, gel artificial skin is most abundant. Gel-based artificial skin has the advantages of being environmentally friendly, highly biocompatible, nontoxic, and similar to human skin in all aspects. Furthermore, sodium alginate, acrylic acid, acrylamide, and other gel materials provide artificial skin with skin-like functions, exempli gratia drive [7], protection [8], signal transmission [9], and other functions. For example, Ye successfully synthesized sandwich-structured hybrid aramid nanofiber–polyvinyl alcohol (PVA)/silver nanowire hydrogels by electrospinning with vacuum-assisted filtration, resulting in various potential applications such as allowing artificial skin to perceive stimulation from external sources [9]. Furthermore, biomimetic morphing materials with anisotropic structures and materials with skin temperature detection have been studied [10,11]. Three-dimensional printing technology has also been applied to artificial skin [12], and the field is moving towards wearables [13]. On the basis of these advantages, numerous studies have been conducted on the practical applications of artificial skin in the past decade.

Although artificial hydrogel skin has many advantages, its disadvantages are evident when compared with real human skin. As human skin ages, human skin can continue to produce oils to prevent drying and cracking. Unavoidably, polymer artificial skin will age by way of chemical decomposition and water loss, resulting in shape shrinkage and mechanical property collapse [14]. Similar self-nourishing behavior occurs in the animal kingdom; for example, the glands in the tail of penguins secrete oil, and the penguins apply the oil to their feathers with their own beaks to ensure that the feathers do not get waterlogged and freeze [15]. Interestingly, based on these natural phenomena, we previously reported a novel concept of self-nourishing, also named as the hollow fiber self-nourishing method. This new concept of self-nourishing was first reported in our previous work about an antiaging bituminous material [16]. Polyvinylidene fluoride (PVDF) hollow fibers were fabricated by a one-step spinning method containing an oily bituminous rejuvenator. The oily liquid can pass through a microporous structure in the shell of the fibers, then penetrate the hollow fiber shell, slowly seeping into the bitumen. This behavior makes the bitumen maintain its original properties for a long time, thus realizing self-nourishing.

Additionally, human skin can repair its wounds using the nutrients delivered by the capillaries. Skin, a natural biomaterial, has the ability to self-heal after damage caused by chemical breakdown or external loads [17]. The self-healing can be achieved at both the microcosmic level (e.g., the skin can moisten itself by secreting sebum) and at the macroscopic level (e.g., healing a cut on the body) [18]. In recent years, the self-healing function has been applied to many fields of artificial skin, such as wearable artificial electronic skin [19], shape memory artificial skin [20], sensing artificial skin [21], and 3D-printed artificial skin [22].

Self-healing polymers can be achieved in three ways: intrinsic reversible bonding, microcapsules, and bionic microvasculars [23]. Artificial skin with self-healing is usually created by intrinsic reversible bonding. In this example, artificial skin is damaged by external stimuli, and next, repolymerization or re-cross-linking by intrinsic reversible bonding occurs to achieve self-healing [24]. However, most repolymerization or re-cross-linking requires some specific conditions to react—for example, temperature and pH [25]. Alternatively, self-healing microcapsules can solve these problems. The healing agent chemistry remains stable in different environmental factors (temperature and pH) because of protection from the shell material [26]. The phase separation between the artificial skin and healing agent simplifies processing, and the healing agent can be easily mixed into artificial skin by the microcapsules with a core–shell structure [27]. When microcracks emerge, the healing agent is released from the microcapsules and the agent wets the microcracks. Furthermore, microcracks achieve self-healing by healing agent curing [28]. Although microcapsules as a self-healing material have some shortcomings that cannot be ignored—such as when the healing agent content is small—the healing capacity is insufficient [29]. Therefore, neither of these methods can fully realize the self-healing of artificial skin. Inspired by biological blood vessels, a method of bionic microvascular for self-healing in artificial skin was explored. Bionic microvasculars have been produced by many manufacturing techniques. They are applied in many ways, such as to adjust the temperature, provide self-nourishment, and for self-healing [30]. Bionic microvasculars can heal more serious wounds than the other methods because their healing agent content is greater. Bionic microvascular technology has been successfully applied in bitumen self-healing and polydimethylsiloxane (PDMS) matrix self-healing [31,32]. Although the bionic microvascular has many advantages, few studies have combined bionic microvasculars with artificial skin.

**Figure 1:**
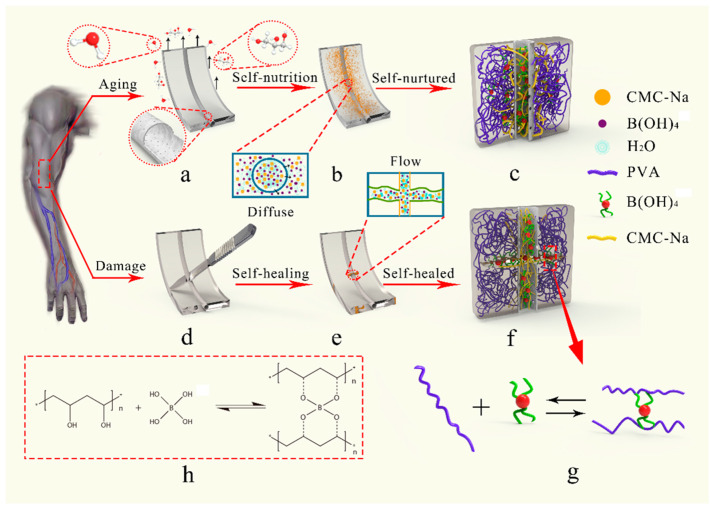
Illustration of the self-nourishing and self-healing function of artificial skin by bionic microvascular. (**a**) The artificial skin’s aging process, volatilize by water and glycerin. The shell of the bionic microvascular has a microporous structure. (**b**) The healing agent released by the microporous structure to achieve self-nourishing function. (**c**) Illustration of the molecular structure of self-nourishing. (**d**) The artificial skin is damaged and wounds appear. (**e**) The healing agent poured by the wounds to achieve self-healing function. (**f**) Illustration of the molecular structure of self-healing. (**g**,**h**) Illustration of the chemical reaction of self-nourishing and self-healing.

In summary, artificial skin that combines self-nourishing and self-healing has led to great interest in bionic microvascular technologies. The main aim of this study was to fabricate and characterize a novel artificial skin comprising a bionic microvascular with self-healing and self-nourishing functions. PVA hydrogel has broadly been used as an artificial skin material [33,34,35]. This study uses a PVA–glycerol–gelatin double network organic hydrogel as the artificial skin matrix. The hydrogel has high mechanical strength because of the strong hydrogen bond formed between the PVA and glycerol (GL), and the gelatin (GEL) increases the toughness and elasticity of the hydrogel to ensure the strength of the artificial skin and the fit of the interface with the body. The continuous bionic microvascular containing a healing agent was prepared by a one-step spinning method using PVDF resin. The bionic microvascular had excellent thermal stability and mechanical property. Because of the microporous structure of the fiber, the fiber had permeability, allowing the healing agent to flow out [16,36]. Figure 1 details the material’s design concept. In normal conditions (Figure 1a–c), the bionic microvascular acts as a reinforcement to strengthen and toughen artificial skin. The healing agent was released by the microporous structure, and the diffusion of water in the gel realizes moisturizing of the healing agent to the matrix. The molecular chain of carboxymethyl cellulose sodium (CMC-Na) physically cross-links with the molecular chain of the matrix to further enhance, toughen, and moisturize the artificial skin. When the artificial skin is severely damaged, the bionic microvascular is broken and the healing agent is released. Boric acid and tetraborate in the healing agent react with PVA at the wound to generate a borate ester bond to heal the skin. CMC-Na also acts as a thickening and healing agent to promote the reaction while forming physical cross-links with the matrix molecular chain, realizing self-healing of the artificial skin (Figure 1d–f). Boric acid and tetraborate in the healing agent reacted with the PVA in the matrix to form the borate ester bond (Figure 1g,h).

## 2. Materials and Methods

### 2.1. Materials

N,N-Dimethylacetimide (DMAC, ≥99.0%), poly(vinylidene fluoride) (PVDF), boric acid (≥99.5%), phosphoric acid (≥85 wt. %), acetate concentrated solution (≥99.8%), poly(vinyl alcohol) (PVA), gelatin (GEL, ≥99.5%), and glycerol (GL, ≥99.5%) were purchased from Aladdin Chemistry Co. Ltd. (Los Angeles, CA, USA). Sodiumhydroxide (NaOH, ≥99.7%) and carboxymethyl cellulose (CMC-Na) were purchased from Sinopharm Chemical Reagent Co. Ltd. (Shangai, China). All reagents were used as received.

### 2.2. Preparation of Artificial Skin Samples

The bionic microvascular was fabricated through a one-step dry–wet method divided into three steps. In the first step, the raw materials for the microvascular were pretreated. PVDF powder was dried in an oven at less than 60 °C for 24 h to completely remove moisture. Amounts of 400 g DMAC solvent and 100 g PVDF powder were poured into a conical flask and sealed immediately. The solvent was stirred and heated to 60 °C in a water bath for 8 h until the PVDF powder completely dissolved in the solvent. This mixture was formed as a casting solution and was vacuum dried at 60 °C for 24 h. The buffer solution used in the core (healing agent) of the bionic microvascular was 0.04 mol/L phosphoric acid and acetic acid. The pH of the buffer solution was controlled to 7, 8, 9, 10, and 11 using NaOH solvent (0.2 mol/L). Quantitative boric acid was added to the buffer solution and stirred to form a stable system. The boric acid content was fixed to test the effect of healing with different pHs, and the resulting pH with the best healing effect. At optimized pH, the healing agents with boric acid contents of 0.5%, 1.0%, 1.5%, 2.0%, 2.5%, and 3.0% were prepared for testing, and the boric acid content with the best effect of healing was obtained. Eventually, 0.5% CMC-Na was added to the system, and the solution was stirred at 40 °C until a uniform viscous liquid was formed.

**Figure 2:**
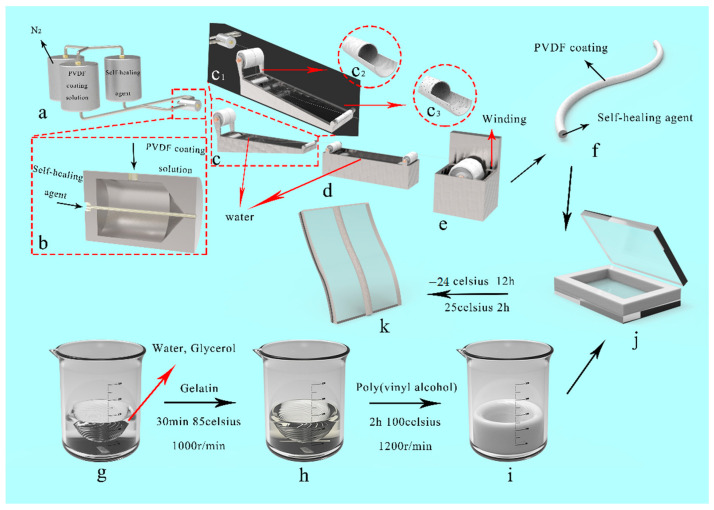
Illustration of preparation of self-nourishing and self-healing artificial skin. (**a**–**e**) Preparation of bionic microvascular. (**c1**–**c3**) Microporous structure preparation of the shell of the bionic microvascular. (**f**) Bionic microvascular. (**g**–**i**) Preparation of the gel. (**j**) The mold for the preparing artificial skin. (**k**) The self-nourishing and self-healing artificial skin.

In the second step, the buffer solution used in the core (healing agent) of the bionic microvascular was added and the solution stored at 25 °C for 24 h. In the last step, the bionic microvascular was fabricated as illustrated in Figure 2.

The casting solution and healing agent were poured into spinning pots at 60 °C (Figure 2a,b). The casting solution was extruded from the outer ring of the coaxial spinneret orifice by N_2_ (0.15 MPa) in a pressure pan. The healing agent was extruded from the centrality ring of the coaxial spinneret orifice by N_2_ (0.2 MPa) in a pressure pan. The extruded bionic microvascular passed through a in 25 °C water coagulation bath (Figure 2c,d). The procedure was used to solidify the bionic microvascular and create the microporous structure shell (Figure 2c1–c3) by elution of DMAC. The solidified bionic microvascular was wrapped around a wheel of twine using a wheel, and the moisture from bionic microvascular surface was removed by a drum wind dryer (Figure 2e). In addition, the two ends of the bionic microvascular were sealed with the inclusion reagent to prevent outflow of the healing agent (Figure 2f).

The matrix of the artificial skin comprised a typical double network: a PVA/GL network and GEL network. As illustrated in Figure 2g–i, distilled water (35 mL) and GL (20 mL) were poured into a beaker and stirred at 500 r/min while heating in an 85 °C water bath. GEL (0.6 g) was then added to the beaker and stirred at 1000 r/min for 30 min. This procedure could loosen the gelatin molecular chain, evenly disperse GEL in the gel system, and help to form a more stable structure within the double network. PVA powder (5 g) was added to the beaker when the solution was a uniform pale yellow, and the solution was heated to 100 °C while stirring at 1200 r/min for 2 h. This process breaks the intramolecular hydrogen bonds of the PVA molecular chain and more intermolecular hydrogen bonds are formed in subsequent reactions. Finally, the air bubbles were eliminated by ultrasonic cleaning. The artificial skin matrix solution was poured into a reaction mold comprising two glass panes (2 mm × 8 cm × 8 cm) and a silicone plate (3 mm × 8 cm × 8 cm). The silicon plate had three dumbbell-shaped grooves, each with a length of 35 mm and a width of 15 mm (Figure 2j). Bionic microvasculars were placed into the mold to form the artificial skin. Artificial skin samples with the bionic microvascular were stored at −24 °C for 12 h and then moved to in a 25 °C oven for 2 h. The prepared artificial skin film was carefully peeled off the mold to avoid tearing (Figure 2k).

### 2.3. Mechanical Properties of Artificial Skin

Artificial skin samples of the bionic microvascular with the healing agent and the empty bionic microvascular were prepared. The gel matrix samples were also prepared. The tensile strength of these samples was measured by using a universal testing machine (Shimadzu AGS-X 50 N, Kyoto, Japan) with a 50 N load and a drawing rate of 1 mm/s at room temperature.

### 2.4. Microporous Structure of Bionic Microvascular

The transverse cross section and shell of the bionic microvascular was observed by SEM (Hitachi TM3030, Tokyo, Japan). The bionic microvascular was quenched in liquid nitrogen (−172 °C). Then, the bionic microvascular was adhered to one side of the sample stage using a conductive double-sided tape. The cross-section surfaces of the bionic microvascular were metal sprayed, and their morphologies were observed at an accelerating voltage of 30 kV.

### 2.5. Self-Nourishing Capability of Artificial Skin

The above samples reacted at 37 °C for 48 h. The tensile strength of these samples was measured by using a universal testing machine (Shimadzu AGS-X 50 N, Kyoto, Japan) with 50 N load and a drawing rate of 1mm/s at room temperature.

### 2.6. Effect of Boric Acid Content on Self-Healing Capability of Artificial Skin

Six sets of artificial skin samples and two sets of gel matrix samples were prepared. A series of different concentrations of borate in the internal bionic microvascular healing agent were chosen, with 0.5%, 1.0%, 1.5%, 2.0%, 2.5% and 3.0%, respectively. A 5 mm wound was made on six artificial skins and one gel matrix by lancet, leaving the other set of gel matrices untreated. These preparations reacted at 37 °C for 24 h. The tensile strength of these samples was measured by using a universal testing machine (Shimadzu AGS-X 50 N, Kyoto, Japan) with 50 N load and a drawing rate of 1mm/s at room temperature.

### 2.7. Effect of pH on Self-Healing Capability of Artificial Skin

Five sets of artificial skin samples and two sets of gel matrix samples were prepared, with different pHs (7, 8, 9, 10, and 11) of the internal bionic microvascular healing agent. A 5 mm wound was made on five artificial skins and one gel matrix by lancet, leaving the other set of gel matrices untreated. Samples were kept at 37 °C for 24 h. The tensile strength was measured by using a universal testing machine (Shimadzu AGS-X 50 N, Kyoto, Japan) with 50 N load and a drawing rate of 1 mm/s at room temperature.

### 2.8. Investigating the Change in Crystallinity of Gel Matrix before and after Healing by X-ray Diffractometer (XRD)

The crystallinity of the gel matrix of the self-nourishing artificial skin is different from the initial state because of the reaction with the healing agent. The crystallinity of the gel matrix at the same location before and after self-nourishing can be compared to determine whether the artificial skin has undergone the self-nourishing reaction. Samples were taken from the artificial skin before and after self-nourishing, respectively, at the same distance from the bionic microvascular location. Samples were lyophilized in a −48 °C freeze dryer until the samples completely lost their moisture. The samples were then ground into powder. The crystallinity of the artificial skin and gel matrix were measured by an XRD (Germany BRUKER D8 DISCOVER, Ettlingen, Germany). The scanning speed was 0.04 °/s, and the diffraction angle (2^θ^) had a range of 5~40°.

### 2.9. Molecular Structure Changes of Gel Matrix before and after Healing by Solid Nuclear Magnetic Resonance Spectrometer (C^13^-NMR)

After the self-healing of the artificial skin, the gel near the wound must have changed its molecular structure because of the reaction with the healing agent. This experiment was conducted to verify that the self-healing behavior of the artificial skin was the result of the reaction between the gel and the healing agent rather than the properties of the gel itself. Samples were taken at the fracture of the artificial skin and the gel matrix that fractured in the tensile test. Samples were lyophilized in a 48 °C freeze dryer until they completely lost their moisture and were then ground into powder. The crystallinity of the artificial skin and gel matrix were measured by a C^13^-NMR (Austria Anton Paar AVANCE NEO 600M, Graz, Austria).

### 2.10. Molecular Structure Changes of Gel Matrix before and after Healing by Fourier Transform Infrared Spectroscopy (FT-IR)

This experiment was conducted to further verify that the self-healing behavior of the artificial skin was the result of the reaction between the gel and the healing agent rather than the properties of the gel itself. Samples were taken at the fracture of the artificial skin that fractured in the tensile test. FT-IR (PerkinElmer Spectrum 100, Waltham, MA, USA) was used to characterize the chemical bands of samples, gel matrix, healing agent, and PVA. FT-IR spectra were recorded in the 400–4000 wavenumber range, adopting a resolution of 4 cm^−1^.

### 2.11. The Appearance of the Artificial Skin Fracture Surface

The appearance of the artificial skin fracture surface was observed by an SEM (Hitachi TM3030, Tokyo, Japan). Samples were taken at the fracture of the artificial skin that fractured in the tensile test and lyophilized in a freeze-drying machine at −48 °C until the samples completely lost moisture. A conductive double-sided tape was used to adhere the sample to one side of stage, then its cross-section surface was metal sprayed while waiting for the SEM test.

### 2.12. Observation of Artificial Skin Self-Healing

Two groups of artificial skin samples (containing a healing agent, a pH = 9, and 2.0% boric acid content) were dyed with methyl violet in the bionic microvascular. One group of artificial skin samples without the healing agent in the bionic microvascular, and another group of gel matrices, were prepared. Wounds with 5 mm size were made on artificial skin with a lancet. When destroying the artificial skin (with a pH = 9, 2.0% boric acid content, and healing agent in the bionic microvascular), the outflow of healing repair fluid was observed and pictures were taken every 25 s. After the damage, it was placed in a 37 °C environment for self-healing for 48 h, and it was taken out for observation and photographed every 12 h. Another group of artificial skin samples (with a pH = 9, 2.0% boric acid content, and a repair agent) was stained with methyl violet and placed at room temperature (25 °C) to observe the healing and take pictures.

## 3. Results and Discussion

### 3.1. Mechanical Properties of the Artificial Skin

**Figure 3:**
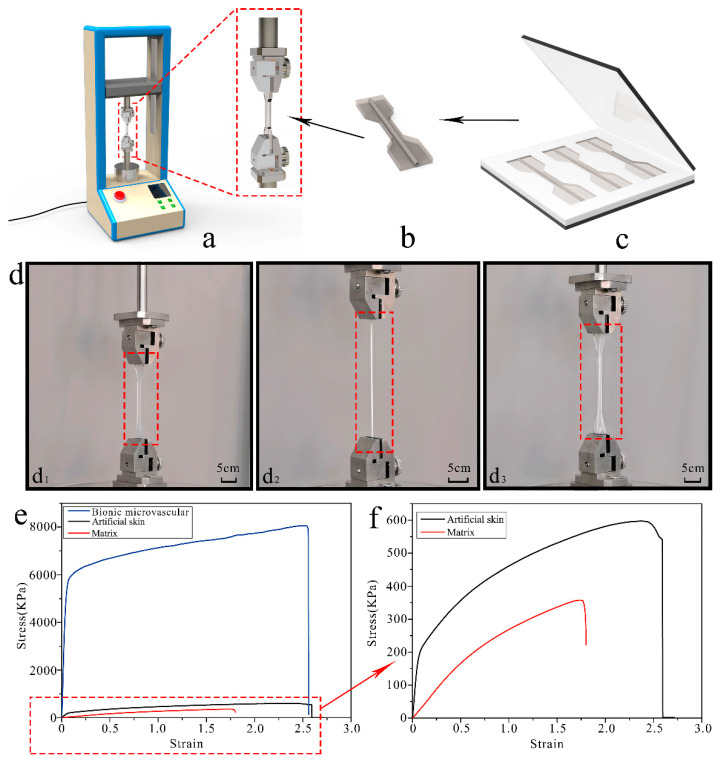
The preparation of artificial skin samples and the mechanical properties of artificial skin. (**a**) Stretching machine for experiments. (**b**) Dumbbell-shaped artificial skin sample. (**c**) The mold for preparing artificial skin samples. (**d**) Comparison of the maximum stretch of a matrix, a bionic microvascular, and artificial skin. (**d1**–**d3**) Matrix, bionic microvascular, and artificial skin. (**e**) The stress–strain curve of a matrix, a bionic microvascular, and artificial skin. (**f**) The stress–strain curve of a matrix and artificial skin.

The mechanical properties of the artificial skin samples were tested by a tensile test at room temperature. Figure 3a illustrates the tensile machine stretching of an artificial skin sample. The artificial skin sample with the microvascular (Figure 3b) was a dumbbell-shaped linear leaf created using a standard mold (Figure 3c). Details of the sample deformation observed during the tensile experiments are shown in Figure 3d and they were used to test and verify the mechanical and tensile properties of the artificial skin samples. Figure 3d1–d3 show the details of the deformation of the gel matrix, bionic microvascular, and artificial skin prior to breaking. The maximum tensile length of the artificial skin was slightly less than that of the bionic microvascular. However, the maximum tensile length of the artificial skin was better than the gel matrix. The incorporation of the bionic microvascular significantly improved the mechanical properties of the artificial skin. Figure 3e,f show the stress–strain curves obtained after processing the data collected during the stretching of the three samples.

The modulus of the bionic microvascular as a reinforcement is much greater than the other samples. The mechanical properties of the artificial skin reinforced by bionic microvascular were much better than those of the gel matrix in all aspects. With the modulus of the artificial skin being greater than that of the gel matrix, the maximum stress of the artificial skin reached 600 KPa. The maximum stress of the gel matrix was only 350 KPa, and the maximum stress of the artificial skin was 1.72 times that of the gel matrix. The maximum strain of the artificial skin reached 2.52 and the maximum strain of the gel matrix was 1.76. The maximum stress of the artificial skin was 1.43 times that of the gel matrix. In summary, the artificial skin enhanced with the bionic microvascular outperformed the gel matrix in all aspects.

This mechanical phenomenon can be explained by the structural analysis of the three samples. First, the intramolecular hydrogen bonds in the PVA molecular chains of the gel matrix were largely broken after stirring at 100 °C, which makes the mixing of PVA, gelatin, and glycerin more uniform. Furthermore, a large amount of intermolecular hydrogen bonds and crystalline regions were formed in the PVA at −25 °C, and a double-network gel structure was formed with gelatin and glycerol. This guarantees the excellent mechanical and tensile properties of the gel matrix in terms of microstructure. PVDF is the raw material of the bionic microvascular; it has excellent stability and weather resistance because its molecular chain has C–F bonds and a helical structure. The short side chains make it impossible to form stronger physical cross-links between the PVDF molecular chains, so there will be relative displacement between molecular chains under the external force. These microscopic factors provide the bionic microvascular with good mechanical tensile properties. Because artificial skin is a composite, the combination of a bionic microvascular as a reinforcement and a gel matrix causes the artificial skin to obtain excellent mechanical properties.

### 3.2. Self-Nourishing Capability of Artificial Skin

**Figure 4:**
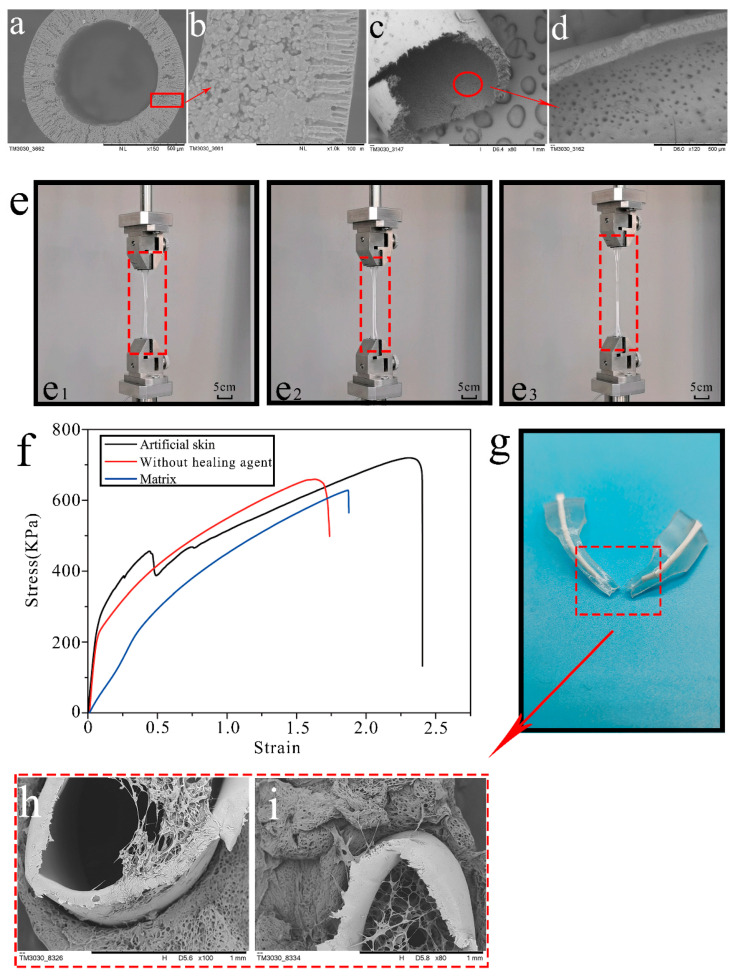
The self-nutrition capability of artificial skin. (**a**,**b**) SEM images of a cross section of a bionic microvascular. (**c**,**d**) SEM images of the shell of a bionic microvascular. (**e**) Comparison of the self-nourishing effect of a matrix without healing agent (no healing agent in bionic microvascular of artificial skin) and artificial skin. (**e1**–**e3**) Matrix without healing agent and artificial skin. (**f**) The stress–strain curve of a matrix with no healing agent and artificial skin. (**g**) Artificial skin sample after tensile fracture. (**h**,**i**) SEM images of the fractured surface of artificial skin.

In this work, a novel artificial skin self-nourishing concept was proposed based on the penetration of an encapsulated agent into the gel matrix. Inspired by capillary bionics, a bionic microvascular was designed with a porous microstructure on its vascular shell. This porous microstructure has the capability of penetrating a self-nourishing agent into an aged gel matrix material, depending on the force of the concentration difference. Figure 4a shows the SEM cross section morphologies of the bionic microvascular shell. The bionic microvascular cross section is a ring shape, with elongated gullies or voids connecting the inside and outside of the bionic microvascular shell. More details are shown in Figure 4b, including a cross section with typical asymmetric structures. The inside of the bionic microvascular shell is a disordered aggregate pore layer, and the outside of the bionic microvascular shell is microporous. The structure is formed because the environment on both sides of the bionic microvascular shell is different during the phase transition. The solvent (DMAC) from the casting solution diffused to the outer coagulation bath (non-solvent water), and the outer coagulation bath diffused to the casting solution. The core material (healing agent) of the bionic microvascular diffusing to the bionic microvascular shell acts as an internal coagulation bath. Thus, the properties and temperatures of the coagulation bath on both sides of the bionic microvascular shell are different, which causes the coagulation speed of the bionic microvascular shell, forming an asymmetric void. The inside of the bionic microvascular shell is observed in Figure 4c,d. The shell is covered with micropores with diameters of approximately 20–25 μm, and the healing agent penetrates outward through the micropores and moisturizes the gel matrix.

Over time, artificial skin exposed to room temperature, water, or glycerol will gradually volatilize, and the artificial skin will shrink and harden. Under an external force, the hardened artificial skin will crack and lose its original function. Fortunately, the bionic microvascular will solve this problem. As artificial skin ages, increased osmotic pressure on both sides of the bionic microvascular shell results in the healing agent in the bionic microvascular leaking into the gel matrix through the micropores of the shell to achieve self-nourishing. In the healing agent, the function of water is moistening the gel matrix and alleviating aging. The PVA crystalline region is broken because boric acid reacts with the PVA of the gel matrix and then the flexibility of the artificial skin is enhanced and aging is alleviated. CMC-Na as the thickening material of the healing agent that seeps through the micropores in the shell of the bionic microvascular and physically cross-links with the gel matrix, which realizes the strengthening and toughening of the artificial skin and enhances the combination of the bionic microvascular and artificial skin matrix.

Three samples were prepared: gel matrix, artificial skin, and artificial skin without healing agent in the bionic microvascular. These three samples were placed in a blast oven at 37 °C for 48 h to fully evaporate the water and glycerol to achieve the aging effect, and then tensile tests were performed at room temperature to test the effect. The deformation of the three samples after aging were clearly observed in the tensile experiments, as shown in Figure 4e, and they can be used to test and verify the mechanical and tensile properties of the artificial skin samples after self-nourishing. Figure 4e1–e3 show details of the deformation of the gel matrix, artificial skin without healing agent in the bionic microvascular, and artificial skin that is about to fracture after being stretched. In terms of tensile strength, the artificial skin outperforms the one without the healing agent in the bionic microvascular, with the gel matrix being the worst in terms of tensile strength.

Figure 4f shows the stress–strain curve obtained after processing the data from the tensile experiments. The maximum stress in the artificial skin without healing agent in the bionic microvascular was 650 KPa and the maximum strain was 1.75; the maximum stress in the gel matrix was 610 Kpa and the maximum strain was 1.85. The stress–strain curve of the artificial skin had two stages: at the beginning of the stretch, when the strain was 0–0.5, the maximum stress of the artificial skin was 475 Kpa, and all mechanical properties of the artificial skin were greater than those of the other two samples. Both groups of artificial skin in the experiment had bionic microvasculars for reinforcement. The only difference was the presence or absence of the healing agent in the bionic microvascular. It is clear that self-nourishing of the artificial skin does slow aging and ensures the mechanical properties of the artificial skin. The stress in the artificial skin was suddenly reduced when the strain reached 0.5 and the bionic microvascular within the artificial skin, which is the main load-bearing component, was broken. The CMC-Na within the healing agent seeped out of the bionic microvascular and created physical cross-linking in the gel matrix. The CMC-Na fixed the relative position of the bionic microvascular in the gel matrix. The fractured artificial skin sample after the tensile experiment (Figure 4g) and the SEM analysis of the lyophilized sample (Figure 4h,i) demonstrated that the CMC-Na penetrated the gel matrix through the microporous structure and physically cross-linked the structure. At this stage, with a strain of 0.5–2.5, the maximum stress in the artificial skin was 700 Kpa and the maximum strain was 2.35. Obviously, the strain of the artificial skin was much greater than the other two samples. For the artificial skin, the modulus of elasticity at this stage was slightly less than that of artificial skin without healing agent in the bionic microvascular; however, its overall mechanical properties in the event of bionic microvascular fracture were much greater than the other two samples. This shows that the self-nourishing of the artificial skin has a significant effect on maintaining its own mechanical properties and extending its service life.

### 3.3. Self-Healing Capability of Artificial Skin

The concept of self-healing artificial skin originates from the self-healing mechanism of human skin after injury. When artificial skin is damaged by an external force, the bionic microvascular is fractured and the healing agent quickly flows out to cover the wound. CMC-Na is the thickening material that fixes other components in the healing agent near the wound and physically cross-links with the gel matrix at the wound to promote wound healing. The borate in the healing agent will react with the PVA molecular chains in the gel matrix to form the borate ester bond to achieve self-healing.

The degree and speed of the reaction between the borate and PVA relates to the effect of self-healing, and the factors affecting the degree and speed are the pH value of the reaction environment and the content of borate in the healing agent. In terms of content, the amount of borate in the healing agent is critical to the healing effect because of the large amount of PVA in the wound of the artificial skin. Alternatively, if there is too much borate in the healing agent, a limited amount of PVA reacts with excess borate quickly. This situation results in carboxyl groups on a single borate not fully reacting with the PVA molecular chains and the inability to form strong chemical cross-links, thereby affecting the self-healing effect of the artificial skin. Alternatively, if there is insufficient borate in the healing agent, an insufficient number of borate ester bond groups are produced, and the self-healing effect of the artificial skin is still not ideal.

**Figure 5:**
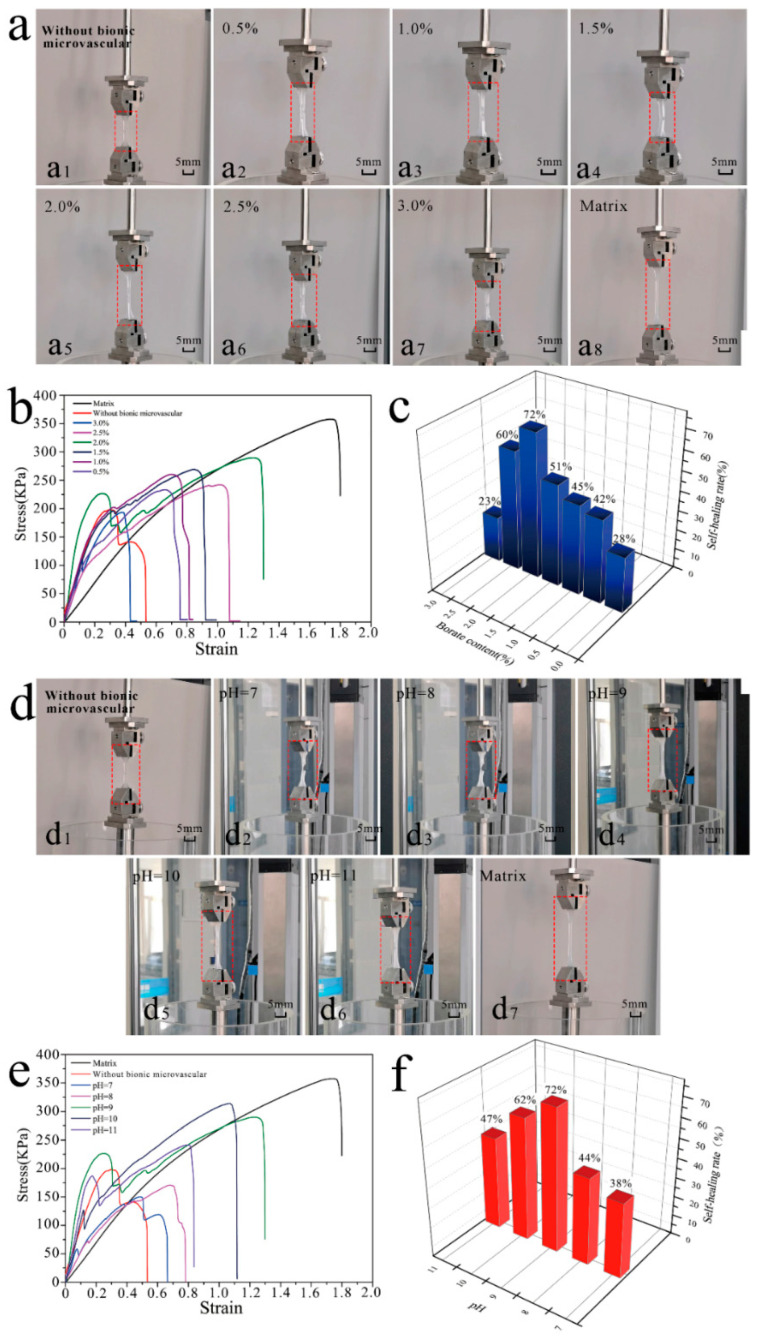
The self-healing capability of artificial skin. (**a**) Comparison of self-healing effect of content of different healing agents. (**a1**) Without bionic microvascular (Tensile test after matrix failure). (**a2**–**a7**) The boric acid content is 0.5%, 1.0%, 1.5%, 2.0%, 2.5%, and 3.0%. (**a8**) Matrix. (**b**) The stress–strain curves of content of different healing agents of artificial skin. (**c**) Relationship between borate content in healing agents and self-healing rate of artificial skin. (**d**) Comparison of self-healing effects of different pH values of healing agents. (**d1**) Without bionic microvascular. (**d2**–**d6**) The pH values are pH = 7, pH = 8, pH = 9, pH = 10, and pH = 11. (**d7**) Matrix. (**e**) The stress–strain curves of different healing agent pH values of artificial skin. (**f**) Relationship between pH in healing agents and self-healing rate of artificial skin.

The images in Figure 5a show the experiment on the effect of borate content in the healing agent on the self-healing efficiency of artificial skin. There were eight groups of samples in the experiment, six of which were artificial skin samples with 0.5%, 1.0%, 1.5%, 2.0%, 2.5%, and 3.0% borate in the bionic microvascular internal (the borate content is used to represent the corresponding artificial skin). All six groups of artificial skin had 5 mm wounds. The other two groups were gel matrix controls, one without wound treatment and the other with the same wounds as the artificial skin samples. Eight sets of samples were placed in a 37 °C environment and made to self-heal for 24 h. Tensile experiments were performed at room temperature to verify the self-healing effects of each group of samples. Figure 5a1 shows the maximum stretch of the gel matrix control group with wounds. Figure 5a2–a7 show the maximum stretch of the artificial skin with 0.5%, 1.0%, 1.5%, 2.0%, 2.5%, and 3.0% healing agent. Figure 5a8 shows the maximum stretch of the gel matrix control group without treatment. The released healing agent had a significant effect on the self-healing of the artificial skin after the bionic microvascular were broken by external forces. The borate content of the healing agent also makes a significant difference in the effect of the self-healing. Figure 5b shows the stress–strain curve after the data processing in the tensile experiment. The self-healing effect of artificial skin does not always increase with the concentration of borate in the healing agent; too high of a concentration of borate has the opposite effect on the self-healing effect. To obtain a clearer relationship between the borate concentration and self-healing effect, the data were processed to obtain the histogram shown in Figure 5c. The graph clearly shows the self-healing efficiency for each borate concentration. When the borate content in the healing agent was 0.5%, the maximum strain of the artificial skin after self-healing was 0.75, and the strain heal rate was approximately 42%; when the borate content in the healing agent was 1.0%, the maximum strain of the artificial skin after self-healing was 0.81, and the strain heal rate was approximately 45%; when the borate content in the healing agent was 1.5%, the maximum strain of the artificial skin after self-healing was 0.92, and the strain heal rate was approximately 51%; when the borate content in the healing agent was 2.0%, the maximum strain of the artificial skin after self-healing was 1.3, and the strain heal rate was approximately 72%. With a further increase in borate content, the self-healing effect of the artificial skin began to decline. When the borate concentration was increased to 2.5%, the maximum tensile strain of the artificial skin was 1.08, reducing the self-healing rate to 60% and reducing the modulus of elasticity. When the borate concentration was increased to 3.0%, the maximum tensile strain of the artificial skin was only 0.41, which was less than the strain (0.52) and modulus of elasticity of the gel matrix control with a wound. In general, the self-healing efficiency of the artificial skin tended to increase and then decrease as the borate content increased. At 0.5–2.0% borate content, the self-healing efficiency of the artificial skin improved with an increasing borate content. In this range, as the borate content increased, the more the gel near the artificial skin wound reacted sufficiently with the borate to form a stronger chemical cross-link, and the self-healing effect was enhanced. When the borate content was greater than 2.0%, there were too many borates near the artificial skin wound, making it impossible for all of the groups on the borates to react completely with the gel; as a result, the chemical cross-linking was not strong enough. Additionally, too many borates can damage the crystalline area and double network structure of the artificial skin, deteriorating the performance of the artificial skin. In summary, the best self-healing artificial skin was achieved at 2.0% borate content in the artificial skin bionic microvascular healing agent.

In terms of pH of the healing agent, the H^+^ break away from hydroxyls on borate is a precondition for borate reacting with PVA, and the alkalinity of the reaction environment affects the dehydrogenation effect of borate. When the pH was low (alkaline), the degree of dehydrogenation of borate in the healing agent is small, and the reactivity of borate is low. Therefore, this situation leads to an insufficient reaction of borate and PVA and the inability to form strong chemical cross-links. When the pH is high, it causes too much dehydrogenated borate in the healing agent. The numerous borates with high reactivity react with PVA at the same time, so that the hydroxyl groups on a single borate group cannot all react with PVA, which will make the chemical cross-links formed by the reaction not strong enough. In addition, the high pH value of the healing agent destroys the overall structure of the artificial skin and reduces its mechanical properties.

The images in Figure 5d show the experiment on the effect of the pH of the healing agent on the self-healing efficiency of artificial skin. There were seven samples in the experiment, five of which were artificial skin samples with a pH of 7, 8, 9, 10, or 11 in the bionic microvascular (the pH was used to represent the corresponding artificial skin). All five groups of artificial skin had 5 mm wounds. The other two samples were gel matrix controls, one without wound treatment and the other with the same wound as the artificial skin samples. The seven samples were placed in a 37 °C environment and reacted for self-healing for 24 h. Tensile experiments were performed at room temperature to verify the self-healing effect. Figure 5d1 shows the details of the maximum stretch of the gel matrix control group with wounds. Maximum stretch values of the artificial skin samples with the healing agent were 7, 8, 9, 10, and 11 (Figure 5d2–d6). Figure 5d7 shows the maximum stretch of the gel matrix control group without treatment. The released healing agent had a significant effect on the self-healing of the artificial skin after the bionic microvasculars were broken by external forces. The pH of the healing agent also had a significant effect on the self-healing. Figure 5e shows the stress–strain curve after the data processing in the tensile experiment. The self-healing effect of artificial skin does not always increase with the pH in the healing agent; a too high pH had the opposite effect on the self-healing. To obtain a clearer relationship between the pH and self-healing effect, the data were processed to obtain the histogram shown in Figure 5f. The graph clearly shows the self-healing efficiency for each pH value. When the pH in the healing agent was 7, the maximum strain of the artificial skin after self-healing was 0.68, and the strain heal rate was approximately 38%; when the pH in the healing agent was 8, the maximum strain of the artificial skin after self-healing was 0.79, and the strain heal rate was approximately 44%; when the pH in the healing agent was 9, the maximum strain of the artificial skin after self-healing was 1.3, and the strain heal rate was approximately 72%. With a further increase in pH, the self-healing effect of the artificial skin began to decline. When the borate concentration was increased to 10, the maximum tensile strain of the artificial skin was 1.11, reducing the self-healing rate to 62%. When the borate concentration was increased to 11, the maximum tensile strain of the artificial skin was 0.85, reducing the self-healing rate to 47%. In general, the self-healing efficiency of the artificial skin tended to increase and then decrease as the pH increased. At pH 7–9, the borate dehydrogenation in the healing agent grows with increasing alkalinity and the gel around the wound reacts more fully with the borate, making the self-healing of the artificial skin more efficient. When the pH increases further, the alkaline environment makes the dehydrogenation of the borate more intense and the self-healing reaction more adequate; however, the excessive reaction and overly alkaline environment severely damages the crystalline zone and gel double network structure of the artificial skin, decreasing the mechanical properties of the artificial skin. In summary, the best self-healing artificial skin was achieved at pH 7 in the artificial skin with bionic microvascular with healing agent.

On the basis of these experiments, the self-healing artificial skin can effectively heal itself after injury. When the pH of the healing agent was 9 and the borate content was 2.0%, the self-healing effect of the artificial skin reached 72%.

### 3.4. The Chemical Mechanisms of Self-Nourishing and Self-Healing Reaction

To further investigate the reaction mechanism of self-nourishing and self-healing of artificial skin, samples were taken at the fracture near the bionic microvascular after the self-nourishing stretching experiment and then characterized by XRD. Samples were also taken at fracture after the self-healing stretching experiment and then characterized by SEM, FT-IR, and C^13^-NMR.

**Figure 6:**
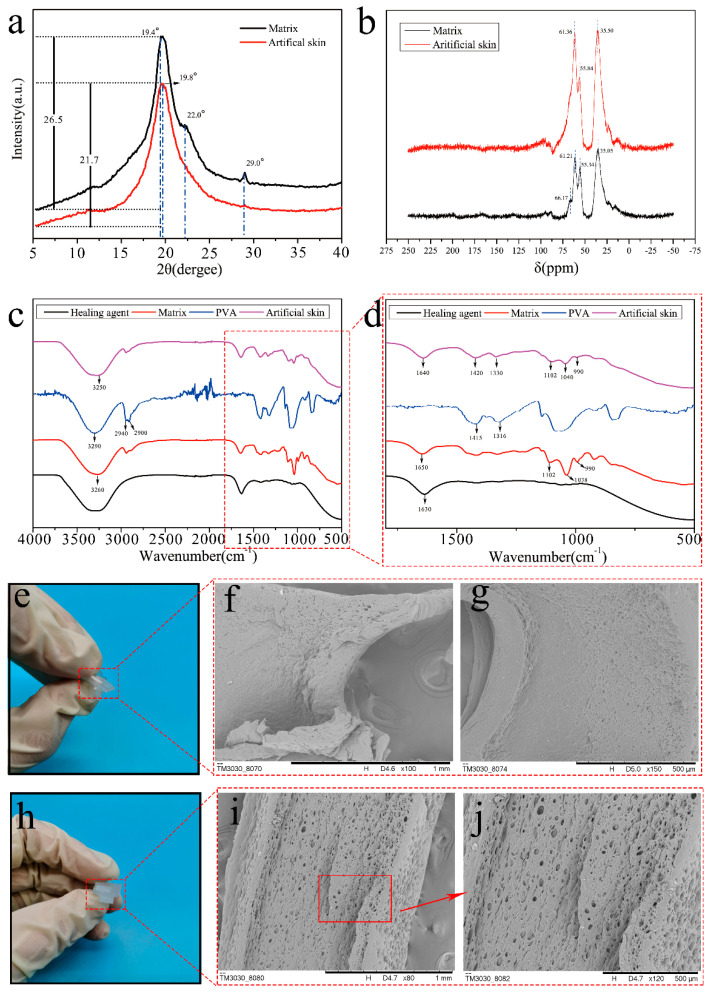
Tests of samples from the fracture surface of artificial skin and matrix samples. (**a**) XRD pattern of artificial skin and matrix. (**b**) C13-NMR curves of artificial skin and matrix. (**c**,**d**) FT-IR curves of artificial skin, matrix, PVA, and healing agent. (**e**) Artificial skin sample after tensile fracture. (**f**,**g**) SEM images of fracture surface of artificial skin. (**h**) Matrix sample after tensile fracture. (**i**,**j**) SEM images of fracture surface of matrix.

Figure 6a shows the XRD pattern of the artificial skin after self-nourishing and the gel matrix. The gel matrix shows two distinct diffraction peaks at 2θ = 19.4° and 2θ = 22.0° that correspond to the orthogonal lattice structure of the semicrystalline PVA [37]. The diffraction peak at 2θ = 19.4° was shifted and weakened in the artificial skin after self-nourishing, and the diffraction peak at 2θ = 22.0° disappeared, indicating the hydroxyl group of PVA reacts strongly with the borate, and the formation of borate ester bonds between them destroys the structure of the crystalline region of PVA.

Figure 6b shows the C^13^-NMR curves of the reacted artificial skin and the gel matrix. Because the artificial skin and gel matrix are mainly made of PVA, the characteristic peak shape is roughly similar to the C^13^-NMR curve of the PVA hydrogel [38]. The addition of gelatin causes a shift in the characteristic peaks, and all characteristic peaks are clustered between 20–80 ppm. PVA molecules have a typical random spatial structure. Except for the methylene signal at 35 ppm, the characteristic peaks at 55 ppm and 61 ppm and the weak characteristic peak at 66 ppm are all methylene carbon resonances. In artificial skin that has undergone a self-healing reaction, the boric acid in the healing agent reacts with the –OH in the PVA, breaking the hydrogen bond. The PVA molecular chain is not bound by the hydrogen bond, which changes its group resonance frequency, thus changing the intensity of the characteristic peak. This further indicates that the healing agent does react with the gel matrix after flowing from the bionic microvascular to heal the artificial skin.

Figure 6c,d show the FT-IR curves of the healing agent, gel matrix, PVA, and self-healing artificial skin. The spectra have similar trends and peak positions; however, there are subtle differences in peak position, peak intensity, and peak shape. For wave numbers in the range of 3100–3700 cm^−1^, all four spectra have distinct peaks that are characteristic of O–H stretching vibrations in the hydroxyl groups. Compared with the gel matrix, the number of hydrogen bonds in the hydrogel increased because of the hydrogen bonding between PVA and gelatin, and the formation of borate ester bonds between PVA and boric acid with the addition of gelatin and the self-healing reaction, which affected the crystallinity of the artificial skin and made the diffraction peaks weaker and shifted to the right. Characteristic absorption bands at 2940 cm^−1^ and 2900 cm^−1^ are C–H. In the spectrum of the gel matrix, PVA, the absorption peak at 1102 cm^−1^ is related to the C–O stretching pattern in PVA, and the peak value of the absorption peak at 1102 cm^−1^ of the artificial skin becomes smaller as the self-healing reaction proceeds. After the self-healing reaction, the artificial skin curve shows several distinct peaks for borates, such as 1330 cm^−1^ and 1420 cm^−1^ (asymmetric stretching vibration peaks for B–O–C complexes). The characteristic peaks of the spectra at 1040 cm^−1^ and 1038 cm^−1^ are related to the vibrational stretching of the C–OH bonds in the artificial skin and matrix, and the C–OH content decreases with self-healing, reducing the characteristic peaks at 1040 cm^−1^.

The cross-sectional microstructure of the freeze-dried self-healing artificial skin and gel matrix was observed by SEM. Figure 6e shows an SEM image of the artificial skin sample after stretching and at fracture. The self-healing artificial skin (Figure 6f,g) has a flatter cross section, with smaller and more sparsely distributed cavities in the cross section. Figure 6h shows an SEM image of the gel matrix sample after stretching and at fracture. As shown in Figure 6i,j, the cross section of the gel matrix with large pores was dense and uniformly distributed, and a typical three-dimensional network structure of the hydrogel was clearly observed. The self-healing resulted in significant changes in the microstructure of the artificial skin.

### 3.5. Observe the Self-Healing Effects of Artificial Skin

**Figure 7:**
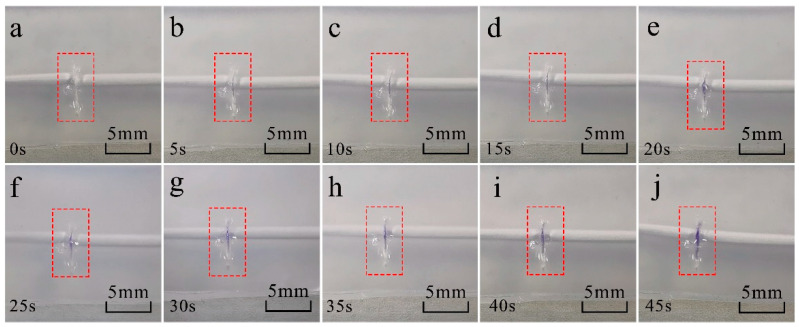
The healing agent (purple) spreading in a formed wound and spilling out over the artificial skin sample surface. (**a**–**j**) Five seconds between each optical image, from 0 s to 45 s.

The artificial skin was cut with a sharp lancet and the flow of the repairing fluid from the bionic microvascular was observed. Figure 7a–f record the outflow of the repair agent 0–45 s after the wound. Because of the wettability of the healing agents, the agents expand along the wound; a narrower wound allows itself to be easily filled with healing agents released from the bionic microvascular. In addition, the overflowing healing agent also wets the top (or bottom) surface of the artificial skin.

The samples were placed for 48 h, observed, photographed every 12 h, and stretched after 48 h to test the self-healing effect. The four samples were the gel matrix bionic microvascular, the artificial skin temperature control group, and the artificial skin group. The self-healing environment temperature of the artificial skin temperature control group was 25 °C, and the self-healing environment temperature of the other three groups was 37 °C (simulation of human body temperature).

**Figure 8:**
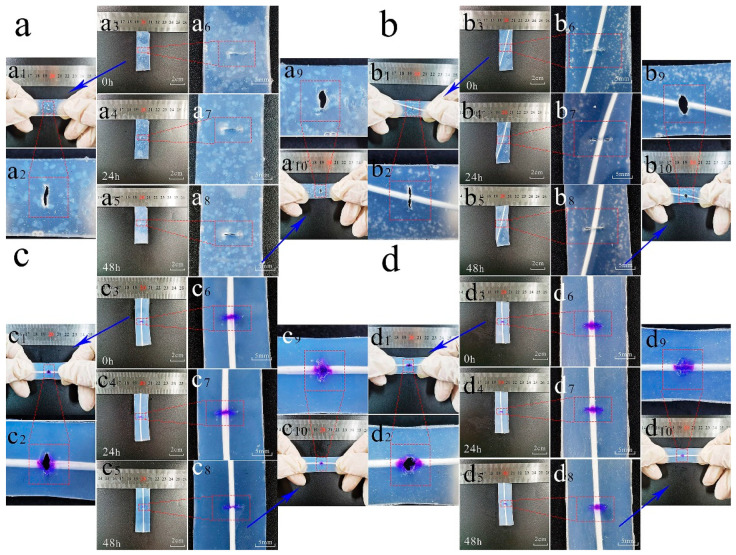
Optical images showing the self-healing process of wound in different conditions within 48 h. (**a**) Self-healing effects of matrix. A 5 mm wound in the center of the matrix, left stationary at 37 °C for 48 h and observed every 12 h ((**a**,**d**) as well). (**a1**–**a10**) Optical images of self-healing effects of the matrix every 12 h. (**b**) Self-healing effects of artificial skin (bionic microvascular without healing agent). (**b1**–**b10**) Optical images of self-healing effects of matrix every 12 h. (**c**) Self-healing effects of artificial skin. A 5 mm wound in the center of the matrix, left stationary at 25 °C for 48 h and observed every 12 h. (**c1**–**c10**) Optical images of self-healing effects of the matrix every 12 h. (**d**) Self-healing effects of artificial skin. (**d1**–**d10**) Optical images of self-healing effects of the matrix every 12 h.

The images in Figure 8a show the gel matrix control group, in which Figure 8a1,a2 show the before images, Figure 8a3–a5 show the overall change from 0–48 h, Figure 8a6–a8 show the change from 0–48 h, and Figure 8a9,a10 show the after images. The following information can be obtained by observing the images. At t = 0 h, the two sides of the wound are tightly adhered. With time, the water and glycerin in the gel on both sides of the wound evaporates. At t = 12 h, the gel on both sides of the wound loses water and shrinks to the extent that it cannot be tightly adhered. At t = 48 h, the wound is still present and the distance between the two sides increases, with a tendency for the wound to expand further. As a result, it is difficult to achieve self-healing with the gel matrix itself.

The images in Figure 8b show the artificial skin control group (without healing agent in the bionic microvascular of the artificial skin), in which Figure 8b1,b2 is the before images, Figure 8b3–b5 show the images of the overall change from 0–48 h, and Figure 8b6–b8 show the images of the change at the wound from 0–48 h, and Figure 8b9,b10 show the after images. The changes were similar to those of the gel matrix control. The only difference was that, because of the different contraction rates of the artificial skin matrix and bionic microvascular, at t = 48 h the gel matrix was more closely fitted near the microvasculature, and the further away from the bionic microvascular, the more severe the separation on both sides of the wound. Although the sample was unable to provide mechanical enhancement to the artificial skin near the wound, the broken bionic microvascular could still promote apposition on both sides of the wound and provide an environment for self-healing.

The images in Figure 8c show the artificial skin temperature control group (self-healing environment at 25 °C), where Figure 8c1,c2 show the before images, Figure 8c3–c5 show the overall change from 0–48 h, Figure 8c6–c8 show the change from 0–48 h at the wound, and Figure 8c9,c10 show the after images. Compared with the other two groups, the healing agent released from the bionic microvascular had the effect of healing the artificial skin, and the wound initially lost its tendency to enlarge and healed. However, because of the low temperature of the healing environment, the reaction activity was relatively slow, allowing incomplete wound healing. In the tensile test at t = 48 h, cracking of the gel matrix around the bionic microvascular was clearly observed, which was caused by the weakness of the gel around the bionic microvascular and the insufficient reaction.

The images in Figure 8d show the artificial skin group, in which Figure 8d1,d2 show the before images, Figure 8d3–d5 show the overall change from 0–48 h, Figure 8d6–d8 show the change from 0–48 h at the wound, and Figure 8d9,d10 show the after images. The wound of the artificial skin was moistened by the healing agent and then was tightly fitted, which provided an environment for the self-healing reaction. By t = 24 h, the wound was largely healed and no longer tended to expand further. At t = 48 h, the wound was further healed and did not crack at the beginning of the tensile test. Temperature was also a factor in the self-healing response.

The following conclusions can be drawn. After damage to the artificial skin, the healing agent released from the broken bionic microvascular can quickly moisten the wound and provide an environment for the wound to heal itself. This plays a crucial role in the self-healing of artificial skin. The broken bionic microvascular also facilitates self-healing by achieving apposition on both sides of the wound. Self-healing of artificial skin is still achieved at room temperature (25 °C), which means that self-healing is achieved by the reaction of the healing agent with the artificial skin, not by the melting and flowing of the artificial skin itself.

## 4. Conclusions

In this experiment, a PVA–GL–GEL double network gel was successfully prepared as a matrix for artificial skin. Smart self-nourishing and self-healing artificial skin was designed by using a PVDF bionic microvascular containing liquid agent with self-nourishing and self-healing characteristics. Through experiments, it was shown that the artificial skin can achieve both self-healing and self-nourishing functions, providing reduced cost and a new direction for the practicality of artificial skin. From the mentioned preliminary results, the following conclusions can be drawn.

In mechanical tensile tests, the maximum stress in the artificial skin (600 KPa) was 1.72 times higher than that of the gel matrix (350 KPa); the maximum strain of the artificial skin (2.52) was 1.43 times higher than that of the gel matrix (1.76). This indicates that the artificial skin—as a composite material with a bionic microvascular as reinforcement—greatly increases the mechanical properties of the artificial skin compared to the gel matrix.

SEM and XRD confirm from a microscopic point of view that the healing agent can achieve self-nourishing of the gel matrix through the microporous structure of the bionic microvascular shell.

Tensile experiments verified this fact macroscopically as well. After aging, the mechanical properties of the artificial skin were superior to those of the gel matrix, as well as to those of the hollow bionic microvascular artificial skin. This shows that the self-nourishing effect of artificial skin does extend its own life.

Would appeared in the artificial skin, it healed itself after 48 h. The effects of the boric acid content and pH value in the healing agent on the self-healing of the artificial skin were also investigated in this paper, and it was finally concluded that the self-healing effect of the artificial skin could reach up to 72% when the pH environment of the healing agent was 9 and the boric acid content was 2.0%.

## Data Availability

Not applicable.
